# The diversity of *Plasmodium falciparum* isolates from asymptomatic and symptomatic school-age children in Kinshasa Province, Democratic Republic of Congo

**DOI:** 10.1186/s12936-023-04528-z

**Published:** 2023-03-20

**Authors:** Shirley V. Simpson, Sabin S. Nundu, Hiroaki Arima, Osamu Kaneko, Toshihiro Mita, Richard Culleton, Taro Yamamoto

**Affiliations:** 1 Programme for Nurturing Global Leaders in Tropical and Emerging Communicable Diseases, Graduate School of Biomedical Sciences, Nagasaki University, Nagasaki, 852-8523 Japan; 2 Department of International Health and Medical Anthropology, Institute of Tropical Medicine (NEKKEN), Nagasaki University, Nagasaki, 852-8523 Japan; 3 Department of Protozoology, Institute of Tropical Medicine (NEKKEN), Nagasaki University, Nagasaki, 852-8523 Japan; 4 Department of Tropical Medicine and Parasitology, Faculty of Medicine, Juntendo University, Tokyo, 113-8421 Japan; 5 Division of Molecular Parasitology, Proteo-Science Centre, Ehime University, Ehime, 790-8577 Japan; 6 Institut National de Recherche Biomédicale (INRB), Kinshasa-Gombe, Democratic Republic of Congo

**Keywords:** *Plasmodium falciparum*, School-age children, Multiplicity of infection, Genetic diversity, Democratic Republic of Congo

## Abstract

**Background:**

Understanding *Plasmodium falciparum* population diversity and transmission dynamics provides information on the intensity of malaria transmission, which is needed for assessing malaria control interventions. This study aimed to determine *P. falciparum* allelic diversity and multiplicity of infection (MOI) among asymptomatic and symptomatic school-age children in Kinshasa Province, Democratic Republic of Congo (DRC).

**Methods:**

A total of 438 DNA samples (248 asymptomatic and 190 symptomatic) were characterized by nested PCR and genotyping the polymorphic regions of *pfmsp1* block 2 and *pfmsp2* block 3.

**Results:**

Nine allele types were observed in *pfmsp1* block2. The K1-type allele was predominant with 78% (229/293) prevalence, followed by the MAD20-type allele (52%, 152/293) and RO33-type allele (44%, 129/293). Twelve alleles were detected in *pfmsp2*, and the 3D7-type allele was the most frequent with 84% (256/304) prevalence, followed by the FC27-type allele (66%, 201/304). Polyclonal infections were detected in 63% (95% CI 56, 69) of the samples, and the MOI (SD) was 1.99 (0.97) in *P. falciparum* single-species infections. MOIs significantly increased in *P. falciparum* isolates from symptomatic parasite carriers compared with asymptomatic carriers (2.24 versus 1.69, adjusted b: 0.36, (95% CI 0.01, 0.72), *p* = 0.046) and parasitaemia > 10,000 parasites/µL compared to parasitaemia < 5000 parasites/µL (2.68 versus 1.63, adjusted b: 0.89, (95% CI 0.46, 1.25), *p* < 0.001).

**Conclusion:**

This survey showed low allelic diversity and MOI of *P. falciparum*, which reflects a moderate intensity of malaria transmission in the study areas. MOIs were more likely to be common in symptomatic infections and increased with the parasitaemia level. Further studies in different transmission zones are needed to understand the epidemiology and parasite complexity in the DRC.

**Supplementary Information:**

The online version contains supplementary material available at 10.1186/s12936-023-04528-z.

## Background

Despite efforts to control and eliminate malaria, the disease continues to threaten the lives of people in 85 countries. Globally, 241 million cases and 627,000 deaths were estimated to have occurred in 2020, of which 96% and 95%, respectively, occurred in the World Health Organization (WHO) African region [1]. *Plasmodium falciparum* remains the most prevalent malaria parasite species in the WHO African region, and accounts for almost all malaria cases [1]. Non-falciparum species are present at a low rate and frequently occur as mixed-species infections with *P. falciparum* [2–6]. Despite a decline in the malaria burden over the last decade, interrupting transmission remains a challenge [7–12].

An efficient malaria vaccine should be part of the arsenal of public health tools. However, the extensive genetic diversity of the parasite is a major obstacle for the acquisition of immunity against malaria, and thus impedes vaccine development [13, 14]. The polymorphic genes encoding the *P. falciparum* merozoite surface proteins 1 and 2 (*Pf*MSP1 and *Pf*MSP2) and the glutamate-rich protein have been used to study allelic diversity and estimate the minimum number of different parasite clones present in malaria infections [15–19]. MSP1 and MSP2 are two key blood stage antigens expressed on the surface of invasive merozoite stage parasites [20] and are dominant targets of the immune response [21]. The major surface protein, MSP1, is a 190-kDa glycosylphosphatidylinositol (GPI)-anchored protein and is encoded by the *msp1* gene located on chromosome 9. It comprises 17 blocks with different degrees of conservation among isolates [18, 21]. Block 2 is the most polymorphic region of the *msp1* gene and can be grouped into three major allelic families (K1 (K), MAD20 (M), and RO33 (R)-types) [22]. Block 2 appears to be under the highest degree of diversifying selection within natural populations [23]. In vaccine development, the incorporation of two main allelic types of block 2 has been shown to strongly protect against *P. falciparum* malaria infections [24], and *Pfmsp1* block 2 combined with a conserved *pfmsp1* block 1 sequence has been shown to enhance the repertoire of MSP-1 block 2 antibody responses [25]. Human anti-MSP1 block 2 antibodies enhance monocyte-mediated phagocytosis of *P. falciparum*, thus protecting against malaria [26].

MSP2 is an ~ 25-kDa GPI-anchored protein abundantly expressed on the merozoite surface. It is encoded by the *msp2* gene located on chromosome 2 and contains 5 blocks, of which block 3 is the most polymorphic [27]. The *msp2* gene has 2 major allelic families, FC27 (F)-type and 3D7 (D)-type [28]. *Pf*MSP2 was found to be a potential vaccine candidate when its two main allelic types were coupled to *Pf*MSP8 (r*Pf*MSP2/8) [29]. *Pf*MSP1 block 2 and *Pf*MSP2 block 3 are mostly targeted by the immune response due to their higher polymorphisms and are associated with protection from clinical malaria [24, 30, 31]. Thus, *pfmsp1* block 2 and *pfmsp2* block 3 may constitute good indicators for measuring malaria transmission intensity and malaria control interventions. The *pfmsp2* gene was previously found to be more genetically diverse than *pfmsp1* in the Democratic Republic of Congo (DRC) [32] and the Republic of Congo and Cameroon [33–35]. MSP2 is considered to be a candidate marker for evaluating parasite virulence [27, 32].

The extent of diversity, estimated in part by polyclonal infection frequency, the number of alleles detected, and the multiplicity of infection (MOI) of *P. falciparum*, constitutes a parameter for measuring the impact of malaria control strategies; and can provide information on malaria transmission intensity that is useful for monitoring and evaluating possible gaps in malaria control interventions [16, 19, 28, 36–39]. These indicators may vary depending on the region due to differences in transmission intensity and malaria control interventions and thus may highlight differences regarding malaria parasite transmission in different public health areas [40, 41].

The Democratic Republic of Congo follows only Nigeria in malaria prevalence in sub-Saharan Africa (SSA) and accounted for 12% of both the estimated global malaria cases and deaths in 2020 [1]. In the DRC, malaria remains a public health problem and is responsible for 44% of all outpatient visits and 22% of deaths among children under five years of age. Approximately 97% of the population lives in stable malaria transmission zones where transmission occurs from 8 to 12 months annually [42].

School-age children are often neglected in malaria control interventions, and thus constitute a reservoir of infections and contribute substantially to malaria transmission [43–48]. In high-transmission settings, approximately 70% of school-age children (5 to 14 years of age) have a higher prevalence of malaria than children under five [45]. Children under five years of age are often at risk of symptomatic malaria, including severe malaria and death, while school-age children mainly carry asymptomatic malaria infections due to acquired immunity as a result of repeated exposure to parasites [49–52]. Thus, studies generally target children under five years of age, while surveys focusing on school-age children are scarce [43, 47]. A report from Senegal showed that the genetic characteristics of parasite populations were different between asymptomatic and symptomatic malaria carriers [53]. Such findings have not yet been established in the DRC.

The genetic diversity of *P. falciparum* in various regions and populations has been extensively studied [18, 32, 36, 39, 54]. However, there is limited information on *pfmsp1* and *pfmsp2* genetic diversity among school-age children in the DRC. Understanding the extent of parasite diversity will provide information on malaria transmission intensity in this population, which will be useful for formulating public health policy. Here, the allelic diversity of *pfmsp1* and *pfmsp2*, the MOI and the prevalence of polyclonal infections in malaria parasites isolated from asymptomatic and symptomatic school-age children in Kinshasa Province, DRC were determined.

## Methods

### Study site and source of samples

Samples for this study were collected as part of a cross-sectional study carried out from October to November 2019 in Kinshasa Province, DRC [48]. The original study was conducted at primary schools and health facilities in the rural area of Mont-Ngafula 2 Health Zone (HZ) and the urban area of Selembao HZ. Mont-Ngafula 2 HZ has been classified as an area at high risk of malaria while Selembao HZ is an area at moderate risk [48, 55]. Briefly, in selected schools, school-age children between 6 and 14 years of age were examined. Children with body temperatures 37.5 °C and below and with no malaria-related symptoms (fever, headache, fatigue, chills, nausea, and vomiting) two weeks before the survey were recruited. In selected health facilities, outpatient children 6 to 14 years of age with fever (body temperatures greater than 37.5 °C) and/or malaria-related symptoms within three days before seeking medical care, and who had not taken anti-malarial drugs were recruited. A total of 438 *P. falciparum*-positive genomic DNA samples were analysed in this study. The details of the study site and sampling procedures have been previously described [48].

### Laboratory analysis

Genomic DNA was extracted from dry blood spots on Whatman 903 filter paper (Whatman plc, UK) using a QIAamp DNA Mini Kit (Qiagen, USA) following the manufacturer’s instructions. The *Plasmodium* species were identified by amplifying the *Plasmodium* mitochondrial cytochrome c oxidase III (*cox3*) gene using a modified protocol described by Nundu et al*.* [48]. In this study, of the 438 *falciparum*-positive samples, 248 asymptomatic infections (162 *P. falciparum* and 86 *P. falciparum* + non-falciparum) and 190 symptomatic infections (144 *P. falciparum* and 46 *P. falciparum* + non-falciparum) were used for PCR analysis [48].

### Genotyping of the *pfmsp1* and *pfmsp2* genes

Nested PCRs were performed to amplify the *pfmsp1* and *pfmsp2* genes using sequence-specific primers as described, with slight modifications [16]. Briefly, primary and secondary PCRs were carried out in a final volume of 20 µL containing 12.5 µL of One Taq® 2X Master Mix (New England Biolabs, Massachusetts, USA). A final concentration of 125 nM of primers was used. The first reaction primer set targeted the conserved regions around block 2 for *pfmsp1* and block 3 for *pfmsp2*. The second reaction used primers specific for allelic families of *pfmsp1* (K-, M-, and R-types) and *pfmsp2* (F- and D-types). Template DNA (2 µL) was used in all first reactions, while 1 µL of amplicon was used for the second reactions. Cycling conditions and primer sets for the respective alleles used in the nested PCRs have been reported elsewhere (Additional file 1: Table S1) [16].

In all reactions, genomic DNA from 3D7 (K-type MSP1 block 2), HB3 (M-type MSP1 block 2), and 7G8 (R-type MSP1 block 2) laboratory strains were used as positive controls [15] and nuclease-free water was used as a negative control. Amplifications were performed on a ProFlex PCR System (Thermo Fisher Scientific, Massachusetts, USA).

All PCR products were analysed by electrophoresis at 100 V for 30 min on 2.5% agarose gels for *pfmsp1* and 2% agarose gels for *pfmsp2*. DNA amplicons were visualized under a UV light transilluminator after post staining with Gel Red® (Biotium, California, USA) solution for 30 min. The fragment sizes were determined using a 100 bp DNA ladder (Takara Bio Inc., Shiga, Japan) and alleles were categorized according to their sizes.

### Data analysis

Categorical variables are presented as proportions and numerical variables by median and interquartile range (IQR) or by means and standard deviation (SD). The *pfmsp1* and *pfmsp2* allelic frequencies were calculated as the proportion of the allele determined for each allele family out of the total alleles detected. The number and size of bands per sample indicated the minimum number of clones in a positive sample. Single infections were samples with only one band and multiple infections were samples with two or more bands. The MOI was determined by calculating the number of different alleles at any locus detected in the sample. The mean MOI was calculated by dividing the respective total number of *pfmsp1* or *pfmsp2* genotypes by the number of positive samples for each marker [15, 34, 56]. Polyclonal infections and MOIs of *pfmsp1* and *pfmsp2* in single-species *P. falciparum* infections were described with proportions and mean (SD), respectively.

The MOIs of *P. falciparum* clones in single and mixed infections were determined for comparison among asymptomatic and symptomatic groups. Evaluations were made to determine the number of *pfmsp1* and *pfmsp2* genotypes in asymptomatic and symptomatic *P. falciparum* monoinfections and in mixed-species infections. The chi-square test was used to compare proportions, Student’s *t test* and ANOVA were used to compare means among categorical groups, and simple and multiple logistic regression with odds ratios (ORs) and 95% confident intervals (95% CIs) were used to establish the associations between independent factors and polyclonal infections. Simple and multiple linear regression with 95% CIs were used to establish the associations between independent factors and the mean MOI. Statistical significance was set at *P* ˂ 0.05. All data were analysed using Stata version 17 (College Station, Texas 77845, USA).

### Ethics statement

The ethics committees of the School of Public Health, University of Kinshasa, DRC (Approval number: ESP/CE/042/2019) and the Institute of Tropical Medicine, Nagasaki University (Approval number: 190110208-2) gave approval for this study [48].

## Results

### General characteristics

For the 438 analysed samples, the frequency of male participants was 50% and the median age was nine years (IQR: 7–11 years) with children aged 6 to 9 years accounting for 60% and children aged 10 to 14 years accounting for 40%. According to the clinical status criteria of school-age children, asymptomatic infections represented 57% of infections, whereas symptomatic infections were 43% and children living in the Mont-Ngafula 2 rural HZ harbour *P. falciparum* parasites represented 60%, versus 40% for those in the Selembao urban HZ (Table 1).

**Table 1:** Baseline characteristics of the children with *P. falciparum*-positive samples

	Sex		Age (years)		Clinical status		Site (HZ)	
Median (IQR)			9 (7–11)					
	Female	Male	6–9	10–14	Asymptomatic	Symptomatic	Rural	Urban
n	219	219	264	174	248	190	261	177
(%)	50.0	50.0	60.3	39.7	56.6	43.4	59.6	40.4

*n* number of samples

### Dynamics of the frequency and diversity of the *pfmsp1* and *pfmsp2* allelic families

Of the 438 samples, 293/438 (67%) for *pfmsp1* and 304/438 (69%) for *pfmsp2* were successfully amplified by PCR. Of 293 *pfmsp1* PCR products, 229 (78%) had at least one K-type allele, 152 (52%) had at least one M-type allele, and 129 (44%) had at least one R-type allele. The proportions of single K-type, M-type, and R-type infections were 30% (87/293), 7% (21/293), and 10% (29/293), respectively. Fifty-three percent of isolates (156/293) harbour polyclonal infections. The frequencies of samples with K/M-type, K/R-type, and M/R-type infections were 19% (56/293), 9% (25/293), and 5% (14/293), respectively. Triple infection of K/M/R-type alleles accounted for 61 samples (21%) (Table 2).

**Table 2:** Allele typing and diversity profiles of *pfmsp1* and* pfmsp2*

Gene	Allele	n (%)	Base pair ranges	No. of genotypes
*msp1*	K1	87 (29.7)	200–350	4
	MAD20	21 (7.2)	200–350	3
	RO33	29 (9.9)	200–300	2
	K1 + MAD20	56 (19.1)		
	K1 + RO33	25 (8.5)		
	MAD20 + RO33	14 (4.8)		
	K1 + MAD20 + RO33	61 (20.8)		
	Total	293		9
*msp2*	FC27	48 (15.8)	300–600	6
	3D7	103 (33.9)	400–700	6
	FC27 + 3D7	153 (50.3)		
	Total	304		12

Of the 304 samples amplified for *pfmsp2*, 201 (66%) had at least one F-type allele, and 256 (84%) had at least one D-type allele. The proportions of single F-type and D-type alleles were 16% (48/304) and 34% (103/304), respectively. Half of the samples, 153/304 (50%), harboured polyclonal infections (Table 2).

A total of 21 allele types were detected in this study: 9 for *pfmsp1* and 12 for *pfmsp2*. For the *pfmsp1* gene, four K-type alleles (approximate fragment sizes 200 bp, 250 bp, 300 bp, and 350 bp), three M-type alleles (approximate fragment sizes 200 bp, 300 bp, and 350 bp), and two R-type alleles (approximate fragment sizes 200 bp and 300 bp) were observed. For the *pfmsp2* gene, six F-type alleles (approximate fragment sizes 300 bp, 350 bp, 400 bp, 450 bp, 500 bp, and 600 bp) and six D-type alleles (approximate fragment sizes 400 bp, 450 bp, 500 bp, 550 bp, 600 bp, and 700 bp) were detected (Table 2).

Alleles of *pfmsp1* were observed at similar distributions in both asymptomatic and symptomatic infections, and in rural and urban settings. Among monoinfections, the K-type was predominant in both asymptomatic and symptomatic infections, and in rural and urban areas. Among double infections, the K/M-type was the most frequent regardless of child clinical status and study area. Triple-infection K/M/R-types in asymptomatic infections accounted for 34/153 (22%), and in rural study areas 40/177 (23%). In symptomatic infections, the K/M/R types accounted for 27/140 (19%), and 21/116 (18%) in the urban areas (Table 3).

**Table 3:** Allelic diversity and frequency of *Pfmsp1* by clinical status and site

Clinical status n (%)			Site n (%)		
Allele	Asymptomatic	Symptomatic	Rural	Urban	
K1	44 (28.8)	43 (30.7)	46 (26.0)	41 (35.3)	
MAD20	10 (6.5)	11 (7.9)	12 (6.8)	9 (7.8)	
RO33	17 (11.1)	12 (8.6)	16 (9.0)	13 (11.2)	
K1 + MAD20	28 (18.3)	28 (20.0)	40 (22.6)	16 (13.8)	
K1 + RO33	13 (8.5)	12 (8.6)	12 (6.8)	13 (11.2)	
MAD20 + RO33	7 (4.6)	7 (5.0)	11 (6.2)	3 (2.6)	
K1 + MAD20 + RO33	34 (22.2)	27 (19.3)	40 (22.6)	21 (18.1)	

*n* number of positive samples

The *pfmsp1* 200 bp K-type, 200 bp M-type, and 200 bp R-type alleles dominated with frequencies above 50% in asymptomatic and symptomatic patients (Additional file 1: Fig. S1).

The distribution of single-allele M-type in rural symptomatic carriers was higher than that in rural asymptomatic carriers, whereas it was higher in urban asymptomatic carriers than in urban symptomatic carriers (Additional file 1: Fig. S2). Likewise, polyclonal infections of K/M-types were more frequent in urban symptomatic carriers than in urban asymptomatic carriers. No significant difference was observed in the distribution of triple infection of K/M/R-types between asymptomatic and symptomatic carriers in either rural or urban areas (Additional file 1: Fig. S3).

The allele frequency of *pfmsp2* differed significantly according to child clinical status. The *pfmsp2* D-type and *pfmsp2* F-type alleles were predominant in asymptomatic carriers and in urban settings, while the combination of the types was more common in symptomatic carriers and rural settings (Table 4).

**Table 4:** Allelic diversity and frequency of *Pfmsp2* by child clinical status and site

Clinical status n (%)			Site n (%)	
Allele	Asymptomatic	Symptomatic	Rural	Urban
FC27	28 (18.3)	20 (13.3)	25 (13.5)	23 (19.3)
IC/3D7	61 (39.9)	42 (27.8)	61 (33.0)	42 (35.3)
FC27 + 3D7	64 (41.8)	89 (58.9)	99 (53.5)	54 (45.4)

For *pfmsp2*, the 400-bp allele was the most prevalent F-type in asymptomatic and symptomatic carriers, whereas the 500-bp D-type allele was most frequent in asymptomatic and symptomatic carriers, with a prevalence of 45% (Table 6). The 600-bp D-type allele was also prevalent with a frequency of 45% in asymptomatic carriers. The allele distribution of *pfmsp2* allelic families is illustrated in Additional file 1: Fig. S4.

In rural and urban areas, the 400-bp FC27 and the 500-bp D-type alleles were the most prevalent, with frequencies above 50% and 40%, respectively (Additional file 1: Fig. S5). The distribution of a single D-type allele among rural asymptomatic carriers was higher than that among rural symptomatic carriers, and single D-type and F-type alleles in urban asymptomatic carriers were higher than those in urban symptomatic carriers. In both rural and urban areas, polyclonal infections of F/D-types were more abundant in symptomatic carriers than in asymptomatic carriers (Additional file 1: Fig. S6).

### Multiplicity of infection (MOI)

Of the 438 DNA samples, 77% (336/438) yielded a positive result for either *msp1* or *msp2* alleles, of which mono-species infection with *P. falciparum* represented 66% (223/336). Polyclonal *P. falciparum* infections accounted for 63% (140/223), of which 59% (114/193) were polyallelic for *pfmsp1* and 49% (98/199) for *pfmsp2*. The overall mean (SD) MOI was 1.99 (0.97)) with the mean (SD) MOI for *pfmsp1* being 1.93 (0.98) and the mean (SD) MOI for *pfmsp2* being 1.67 (0.78) (Table 5).

**Table 5:** Polyclonal infections and MOIs of *pfmsp1* and *pfmsp2* in single-species *P. falciparum* infections

Gene	Monoclonal infections n/N (%)	Polyclonal infections n/N (%)	MOI	SD
*msp1*	79/193 (40.9)	114/193 (59.1)	1.93	0.98
*msp2*	101/199 (50.7)	98/199 (49.3)	1.67	0.78
Total	83/223 (37.2)	140/223 (62.8)	1.99	0.97

*N* number of samples genotyped for *pfmsp1* and *pfmsp2*, *n* number of samples with single or multiple infections for *msp1* or *msp2*

### Polyclonal infections and MOIs of combined *pfmsp1* and *pfmsp2* among school-age children

*Plasmodium falciparum* isolates from symptomatic children had harboured significantly more polyclonal infections than those from asymptomatic children (74% versus 49%, OR: 2.96 (95% CI 1.69, 5.19), *p* < 0.001) when using bivariate analysis but this was not significant after adjustment by density (*p* > 0.05). Higher-density infections with more than 10,000 parasites/µL (88% versus 69%, OR: 3.37 (95% CI 1.30, 8.68, *p* = 0.012)) had significantly more polyclonal infections than infections with a parasite density below 5,000 parasites/µL when using bivariate analysis but this was not significant after adjustment for clinical form of malaria (*p* > 0.05). No significant association of age, sex, or location with polyclonal infections was found (Table 6).

**Table 6:** Polyclonal infections by age, sex, child clinical status, parasite density, and study site

Variables	Number	Polyclonal infections		aOR (95% CI)	*p* value
		n (%)	cOR (95% CI)		
Age (years)					
6–9	144	93 (64.6)	1	1	
10–14	79	47 (59.5)	0.81 (0.46, 1.42)	0.75 (0.31, 1.84)	0.53
Sex					
Female	114	71 (62.3)	1	1	
Male	109	69 (63.3)	1.04 (0.61, 1.80)	1.03 (0.44, 2.42)	0.95
Clinical status					
Asymptomatic	100	49 (49.0)	1	1	
Symptomatic	123	91 (74.0)	2.96 (1.69, 5.19)	2.08 (0.82, 5.28)	0.12
Site					
Urban	101	60 (59.4)	1	1	
Rural	122	80 (65.6)	1.30 (0.75, 2.25)	1.29 (0.54, 3.08)	0.56
Density (parasites/µL)					
< 5000	65	45 (69.2)	1	1	
5000–10,000	21	18 (85.7)	2.67 (0.70, 10.09)	2.34 (0.60, 9.03)	0.22
> 10,000	60	53 (88.3)	3.37 (1.30, 8.68)	2.27 (0.78, 6.63)	0.13

*n*: number of positive samples, *CI* confidence interval, *cOR* crude odds ratio, *aOR* adjusted odds ratio

^*^Wald test

*Plasmodium falciparum* isolates from symptomatic children had harboured significantly higher MOIs than those from asymptomatic children (2.24 versus 1.69, crude b: 0.55 (95% CI 0.30, 0.79, *p* < 0.001)) when using bivariate analysis, and this remained significant after adjustment for density (adjusted b: 0.36 (95% CI 0.01, 0.72, *p* = 0.046)). Infections with densities of 5000–10,000 parasites/µL (2.24 versus 1.63, crude b: 0.61 (95% CI 0.12, 1.10, *p* = 0.014)) and those with over 10,000 parasites/µL (2.68 versus 1.63, crude b: 1.06 (95% CI 0.71, 1.39, *p* < 0.001)) had significantly higher mean MOIs than those with parasite densities below 5,000 parasites/µL when using bivariate analysis, and this remained significant after adjustment for the clinical form of malaria (adjusted b: 0.52 (95% CI: 0.03, 1.01, *p* = 0.038) and adjusted b: 0.89 (95% CI 0.46, 1.25, *p* < 0.001), respectively)). No significant association of sex, age, or location with MOI was found** (**Table 7).

**Table 7:** Multiplicity of infection by age, sex, child clinical status, and study site

Variables	Number	MOI (95% CI)	Crude b (95% CI)	Adjusted b (95% CI)	*p* value^*^
Age (years)					
6–9	144	2.03 (1.87, 2.20)	0	0	
10–14	79	1.91 (1.71, 2.11)	− 0.12 (− 0.39, 0.14)	− 0.28 (− 0.62, 0.06)	0.11
Sex					
Female	114	1.99 (1.81, 2.18)	0	0	
Male	109	1.99 (1.81, 2.17)	− 0.01 (− 0.26, 0.26)	− 0.14 (− 0.46, 0.18)	0.39
Clinical status					
Asymptomatic	100	1.69 (1.53, 1.85)	0	0	
Symptomatic	123	2.24 (2.05, 2.42)	0.55 (0.30, 0.79)	0.36 (0.01, 0.72)	**0.046**
Site					
Urban	101	2.00 (1.80, 2.20)	0	0	
Rural	122	1.98 (1.82, 2.15)	− 0.02 (− 0.27, 0.24)	− 0.06 (− 0.39, 0.26)	0.70
Density(parasites/µL)					
< 5000	65	1.63 (1.39, 1.86)	0	0	
5000–10,000	21	2.24 (1.92, 2.56)	0.61 (0.12, 1.10)	0.52 (0.03, 1.01)	**0.038**
> 10,000	60	2.68 (2.41, 2.95)	1.06 (0.71, 1.39)	0.89 (0.46, 1.25)	** < 0.001**

*CI* confidence interval, *b* coefficient

^*^two-tailed *t test*

Bolded *p value* indicates values lower than 0.05

### Multiplicity of infection in mono-species *P. falciparum* and mixed-species *P. falciparum*/*P. malariae*, *P. falciparum*/*P. ovale* and *P. falciparum*/*P. malariae*/*P. ovale* infections

Overall, more than one genotype per infection was observed in the majority of infections. The presence of *Plasmodium ovale* and/or *Plasmodium malariae* in coinfections with *P. falciparum* was associated with increased MOI (*p* = 0.001) and frequencies of polyclonal infections (*p* = 0.016) compared to single-species infection of *P. falciparum*. This significantly increased the MOI of *msp1* (p = 0.002) and polyclonal infections in *msp2* (p = 0.017) (Table 8).

**Table 8:** Multiplicity of infection of *pfmsp1* and *pfmsp2* in monoinfections of *P. falciparum* and mixed-species-infection of *P. falciparum*/*P. malariae*, *P. falciparum*/*P. ovale* and *P. falciparum*/*P. malariae*/*P. ovale* isolates

Gene	*P. falciparum*	*P. falciparum/P. malariae*	*P. falciparum/P. ovale*	*P. falciparum/P. malariae/P. ovale*	*p* value^*^
Overall					
msp1/msp2					
Polyclonal infections	62.8	77.8	85.2	78.1	**0.016**
MOI	1.99	2.20	2.74	2.16	**0.001**
msp1					
Polyclonal infections	59.1	68.7	79.2	71.4	0.14
MOI	1.93	2.15	2.75	2.07	**0.002**
msp2					
Polyclonal infections	49.3	64.6	65.4	74.2	**0.017**
MOI	1.67	1.71	1.85	1.87	0.41
Asymptomatic infections					
msp1/msp2					
Polyclonal infections	49.0	76.2	66.7	79.2	**0.004**
MOI	1.69	2.12	2.33	2.13	**0.004**
msp1					
Polyclonal infections	46.1	75.0	85.7	76.2	**0.002**
MOI	1.66	2.17	2.57	2.14	**0.001**
msp2					
Polyclonal infections	33.3	56.7	33.3	78.3	**0.001**
MOI	1.38	1.59	1.44	1.83	**0.008**
Symptomatic infections					
msp1/msp2					
Polyclonal infections	74.0	83.3	94.4	75.0	0.26
MOI	2.24	2.5	2.94	2.25	0.05
msp1					
Polyclonal infections	70.2	50.0	76.5	57.1	0.40
MOI	2.16	2.08	2.82	1.86	0.10
msp2					
Polyclonal infections	60.9	90.1	82.3	62.5	0.09
MOI	1.89	2.09	2.06	2.00	0.74

^*^The chi-square test was used for polyclonal infections, and the *ANOVA*-*test* was used to compare mean MOI

Bolded *p values* indicate values lower than 0.05

Stratifying for child health status, the presence of *P. ovale* and/or *P. malariae* in coinfections with *P. falciparum* significantly increased the MOIs and polyclonal infections among asymptomatic carriers (*p* < 0.05), while no significant difference was found among symptomatic carriers (Table 8).

## Discussion

In the DRC, school-age children are often neglected in malaria prevalence surveys, and therefore benefit less from malaria prevention measures than children under five years of age and pregnant women [57]. School-age children contribute to transmission because they may represent a substantial malaria parasite reservoir [14, 44, 46, 48, 58], and thus represent a gap in the formulation of malaria containment strategies. Less attention has been given to investigating the genetic diversity of *P. falciparum* isolates in the DRC compared to other malaria endemic countries.

This is the first study to provide a detailed assessment of allelic diversity and multiplicity of *P. falciparum* infections among the underserved school-age group in the DRC using the polymorphic regions of the genes coding for MSP1 and MSP2.

The purpose of this study was to evaluate the extent of *P. falciparum* diversity, including polyclonal infection frequency, the number of alleles detected, and the MOI among asymptomatic and symptomatic individuals living in rural and urban areas of Kinshasa Province, DRC. The findings of this study showed that no allelic families of *pfmsp1* (K-, M-, and R-types) or *pfmsp2* (F- and D-types) were exclusively restricted to either rural or urban settings, as well as asymptomatic or symptomatic carriers, although the frequency of some alleles was more common at one site compared to another, in addition to differences based on child health status.

The *pfmsp2* gene was found to be genetically more diverse than the *pfmsp1* gene in the study areas, in support of the findings of the majority of studies conducted in Africa [15, 33–35, 59]. This confirms that in many malaria-endemic African countries, polymorphic *pfmsp2* allelic families may be circulating at higher frequencies than those for *pfmsp1*. MSP1 block 2 has been proposed to be most considerably exposed to host immune selective pressure based on the stable K-M-R frequency distribution in endemic regions [24]. Escaping with 3 major alleles of MSP1 block 2 may have an advantage compared to MSP2, which has only 2 major alleles. This may stimulate diverse MSP2 repeat number polymorphisms.

Symptomatic children have been shown to have more polyclonal infections than asymptomatic children and those polyclonal infections increased with the level of parasitaemia regardless of clinical status. In Côte d’Ivoire, Gnagne et al*.* [60] found significant association of polyclonal infections measured by both *pfmsp1* and *pfmsp2* with an increase in parasitaemia but not with clinical status, while in southern Benin, Lagnika et al. [61] found that polyclonal infections were more prevalent among symptomatic malaria carriers than asymptomatic carriers. This may be due to the low power to amplify minor alleles in the samples with low levels of parasitaemia. Thus, further investigations are needed to establish the impact of clinical status and parasitaemia on the multiplicities of infection.

This study showed a generally lower MOI than has been previously reported in the DRC, Cameroon, and Republic of Congo [32, 34, 35]. Conversely, this study showed a higher MOI than that in reports from Grande Comore Island, Côte d’Ivoire, and Gabon [59–63], whereas it was similar to an MOI reported in the Republic of Congo by Mayengue et al*.* [33]. These observations may indicate differences in malaria-related seasonal and transmission settings and improvements in country-specific malaria intervention measures. Alternatively, this may be due to the heterogeneity of *P. falciparum* transmission, which differs from one area to another, resulting in heterogeneous malaria transmission rates throughout the area [48, 62, 63], as MOI measures malaria transmission or host acquired immunity or severity level [32, 52, 62]. For instance, some reports from the DRC and other countries showed a positive correlation of the MOI with malaria recrudescence [32] and parasitaemia level [19, 33, 52] and a negative correlation between the MOI and acquired immunity level [64]. Conversely, other studies did not find a significant association of the MOI with either parasitaemia or clinical status [35, 63]. However, the studies that established the relationship of the MOI with either parasitaemia or clinical status did not associate them (parasitaemia and clinical status) to minimize confounding factors. There is a need for further investigations to consider parasitaemia and clinical status together and their possible association with the MOI. In Gabon and Grande Comore Island, it has been shown that the MOI may vary by region [61, 64] and decrease due to intervention measures and after the implementation of artemisinin-based combination therapy (ACT) [62, 63].

This study did not find a significant difference in the overall MOI, *pfmsp1* MOI, or *pfmsp2* MOI between the two study areas (rural versus urban), although a previous report from the same areas and time period showed that the odds of the transmission of *Plasmodium* infections were fivefold higher in rural versus urban areas [48]. However, the mean MOI in the rural area was slightly, but not significantly, higher than that in the urban area. In Senegal, Ndiaye et al*.* [65] showed that the MOI was significantly higher in a rural area than in an urban setting. To date, the majority of studies have established: i) a correlation between the genetic diversity of *P. falciparum* populations and the intensity of transmission in malaria-endemic areas, ii) that *P. falciparum* genetic diversity is higher in hyperendemic areas than in low-endemic settings [41, 65–67], and iii) that polyclonal infection prevalence decreases with a reduction in malaria transmission [67–72]. These findings suggest that *pfmsp1* and *pfmsp2* allelic diversities are not influenced by the level of transmission between the two zones. However, a lower number of polyclonal infections and a lower MOI in Selembao urban HZ may reflect the moderate malaria transmission level in that area [48, 55], which agrees with studies conducted in hypoendemic areas [36, 48, 72] compared to the higher number of polyclonal infections found in Mont-Ngafula 2 rural HZ, which reflects its malaria hyperendemicity [48, 55], as shown in areas with hyperendemic malaria in Africa [48, 73–76]. Selembao has been described earlier as a peri urban area [55] and may not be a true representation of an urban environment of Kinshasa. Hence, spatiotemporal analyses are needed in different provinces and in different areas within and among the DRC provinces to characterize and better understand the parasite complexity in the DRC.

These findings showed the predominance of single *pfmsp1* K- and M-type alleles in *P. falciparum* mono-species infections and that of the K/M/R triple *pfmsp1* alleles in mixed-species infections with *P. falciparum* regardless of malaria clinical status. For the *pfmsp2* gene, the D-type allele was more frequent in *P. falciparum* mono-species infections, while the F/D-type double infection was more frequent in mixed-species infections in both symptomatic and asymptomatic carriers. The diverse distribution of *pfmsp1/2* alleles within *P. falciparum* mono- and mixed-species infections may be explained based on findings from experimental [76, 77] and clinical [78–80] studies that have shown cross-species immunity within hosts coinfected by multiple *Plasmodium* species. This situation may be influenced by within-host competition between species independent of immunity. Tang et al*.* [81] showed that a mixed infection of two rodent malaria parasite species could increase the severity and parasite densities in the mouse host. Studies are needed to assess the immunity-independent influence of mixed-infections of human *Plasmodium* within hosts.

In southern Benin, it has been shown that the prevalence of polyclonal infections and MOIs as measured by polymorphisms in *pfmsp1* and *pfmsp2* were significantly lower in *P. falciparum*/*P. malariae-*coinfected asymptomatic carriers compared to those with a single-species infection of *P. falciparum,* and it was suggested that a decrease in genetic diversity and complexity of infection occurred in cases of coinfection [80]. Conversely, this study showed an increase in polyclonal infections and MOIs as measured by polymorphisms in *pfmsp1* and *pfmsp2* in infections with *P. ovale* and/or *P. malariae* coinfected with *P. falciparum* among asymptomatic subjects, although it was not significantly different among symptomatic subjects. Further studies are needed to provide more information on the consequences of within-host competition in mixed-species infections based on malaria clinical status.

This study found that for *pfmsp1*, an allele size of approximately 200 bp dominated for all K-, M-, and R-types, regardless of the presence of symptoms and the rural and urban settings. This finding was also shown in Cameroon [18], Gabon [59, 83], Burkina Faso [15], Benin [82], and the Republic of Congo [33]. For *pfmsp2*, the 400-bp F-type allele and the 500-bp D-type allele dominated, regardless of the presence of symptoms and the rural and urban settings. Among the F-type *pfmsp2* alleles, the 400-bp allele has also been shown to be frequent in the Republic of Congo [33] and Burkina Faso [15] whereas the 500-bp allele was more frequent in Côte d’Ivoire [59], the 600-bp allele in Gabon [59], and the 700-bp allele in Benin [82]. Among the D-type *pfmsp2* alleles, the 300-bp allele was more frequent in the Republic of Congo [33], Côte d’Ivoire [59], and Burkina Faso [15] while the 400-bp allele was more frequent in Gabon [59] and the 700-bp allele was more frequent in Benin [82]. Future sequence analysis of genetic polymorphisms could confirm the similarities of these allelic families with those in other African countries [28, 34, 60, 81–84].

## Conclusion

The allelic diversities and MOIs of *P. falciparum* isolates from asymptomatic and symptomatic school-age children were low in the study areas of Kinshasa Province, DRC. MOIs were more likely to be present in symptomatic infections and increased with the parasitaemia level. There is a need to conduct a countrywide study on the genetic diversity of *P. falciparum* in different transmission zones to provide a clear picture of parasite diversity and to serve as a baseline for determining the impact of malaria interventions on parasite genetic diversity in the country.

## Supplementary Information

**Additional file 1: Figure S1.** Allelic frequencies in *pfmsp1* block 2 in asymptomatic (a) and symptomatic (b) carriers and in both rural and urban areas**. Figure S2.** Allelic frequencies in *pfmsp1* block 2 in rural (a) and urban (b) areas. **Figure S3.** Allelic frequencies in *pfmsp1* block 2 in asymptomatic and symptomatic infections stratified by rural (a) and urban (b) areas. **Figure S4.** Allelic frequencies in *pfmsp2* in asymptomatic (a) and symptomatic (b) carriers. **Figure S5.** Allelic frequencies in *pfmsp2* in rural (a) and urban (b) areas. **Figure S6.** Allelic frequencies in *pfmsp2* in asymptomatic and symptomatic carriers stratified rural (a) and urban (b) areas

## Data Availability

The datasets used and/or analysed during the current study are available from the first author (SVS) and corresponding author (SSN) upon request.

## References

[1] Olapeju B, Choiriyyah I, Lynch M, Acosta A, Blaufuss S, Filemyr E (2018). Age and gender trends in insecticide-treated net use in sub-Saharan Africa: a multi-country analysis. Malar J.

[2] Doctor SM, Liu Y, Anderson OG, Whitesell AN, Mwandagalirwa MK, Muwonga J (2016). Low prevalence of *Plasmodium malariae* and *Plasmodium ovale* mono-infections among children in the Democratic Republic of the Congo: a population-based, cross-sectional study. Malar J.

[3] Asua V, Tukwasibwe S, Conrad M, Walakira A, Nankabirwa JI, Mugenyi L (2017). *Plasmodium* species infecting children presenting with malaria in Uganda. Am J Trop Med Hyg.

[4] Sitali L, Miller JM, Mwenda MC, Bridges DJ, Hawela MB, Hamainza B (2019). Distribution of *Plasmodium* species and assessment of performance of diagnostic tools used during a malaria survey in Southern and Western Provinces of Zambia. Malar J.

[5] Yerlikaya S, Campillo A, Gonzalez IJ (2018). A systematic review: performance of rapid diagnostic tests for the detection of *Plasmodium knowlesi*, *Plasmodium malariae*, and *Plasmodium ovale* monoinfections in human blood. J Infect Dis.

[6] Amanfo SA, Mduluza T, Midzi N, Cavanagh DR, Mutapi F (2016). Seroepidemiology of *Plasmodium* species infections in Zimbabwean population. Malar J.

[7] Ranson H, Lissenden N (2016). Insecticide resistance in African *Anopheles* mosquitoes: a worsening situation that needs urgent action to maintain malaria control. Trends Parasitol.

[8] Bhatt S, Weiss DJ, Cameron E, Bisanzio D, Mappin B, Dalrymple U (2015). The effect of malaria control on *Plasmodium falciparum* in Africa between 2000 and 2015. Nature.

[9] Snow RW, Sartorius B, Kyalo D, Maina J, Amratia P, Mundia CW (2017). The prevalence of *Plasmodium falciparum* in sub-Saharan Africa since 1900. Nature.

[10] Benelli G, Jeffries CL, Walker T (2016). Biological control of mosquito vectors: past, present, and future. Insects.

[11] Nkumama IN, O'Meara WP, Osier FHA (2017). Changes in malaria epidemiology in Africa and new challenges for elimination. Trends Parasitol.

[12] Rabinovich RN, Drakeley C, Djimde AA, Hall BF, Hay SI, Hemingway J (2017). malERA: an updated research agenda for malaria elimination and eradication. PLoS Med.

[13] Abamecha A, El-Abid H, Yilma D, Addisu W, Ibenthal A, Bayih AG (2020). Genetic diversity and genotype multiplicity of *Plasmodium falciparum* infection in patients with uncomplicated malaria in Chewaka district. Ethiopia Malar J.

[14] Gueye NSG, Ntoumi F, Vouvoungui C, Kobawila SC, Nkombo M, Mouanga AM, Deibert J (2018). *Plasmodium falciparum* merozoite protein-1 genetic diversity and multiplicity of infection in isolates from Congolese children consulting in a pediatric hospital in Brazzaville. Acta Trop.

[15] Somé AF, Bazié T, Zongo I, Yerbanga RS, Nikiéma F, Neya C (2018). *Plasmodium falciparum msp1* and *msp2* genetic diversity and allele frequencies in parasites isolated from symptomatic malaria patients in Bobo-Dioulasso. Burkina Faso Parasit Vectors.

[16] Snounou G, Zhu X, Siripoon N, Jarra W, Thaithong S, Brown KN (1999). Biased distribution of msp1 and msp2 allelic variants in *Plasmodium falciparum* populations in Thailand. Trans R Soc Trop Med Hyg.

[17] Mohammed H, Mindaye T, Belayneh M, Kassa M, Assefa A, Tadesse M (2015). Genetic diversity of *Plasmodium falciparum* isolates based on *msp-1* and *msp-2* genes from Kolla-Shele area, Arbaminch Zuria District, southwest Ethiopia. Malar J.

[18] Apinjoh TO, Tata RB, Anchang-Kimbi JK, Chi HF, Fon EM, Mugri RN (2015). *Plasmodium falciparum* merozoite surface protein 1 block 2 gene polymorphism in field isolates along the slope of mount cameroon: a cross - sectional study. BMC Infect Dis.

[19] Chen JT, Li J, Zha GC, Huang G, Huang ZX, Xie DD (2018). Genetic diversity and allele frequencies of *Plasmodium falciparum msp1* and *msp2* in parasite isolates from Bioko Island. Equatorial Guinea Malar J.

[20] Holder AA, Blackman MJ, Burghaus PA, Chappel JA, Ling IT, McCallum-Deighton N (1992). A malaria merozoite surface protein (MSP1)-structure, processing and function. Mem Inst Oswaldo Cruz.

[21] Woehlbier U, Epp C, Kauth CW, Lutz R, Long CA, Coulibaly B (2006). Analysis of antibodies directed against merozoite surface protein 1 of the human malaria parasite *Plasmodium falciparum*. Infect Immun.

[22] Takala SL, Escalante AA, Branch OH, Kariuki S, Biswas S, Chaiyaroj SC (2006). Genetic diversity in the Block 2 region of the merozoite surface protein 1 (MSP-1) of *Plasmodium falciparum*: additional complexity and selection and convergence in fragment size polymorphism. Infect Genet Evol.

[23] Holder AA, Blackman MJ (1994). What is the function of MSP-I on the malaria merozoite?. Parasitol Today.

[24] Conway DJ, Cavanagh DR, Tanabe K, Roper C, Mikes ZS, Sakihama N (2000). A principal target of human immunity to malaria identified by molecular population genetic and immunological analyses. Nat Med.

[25] Cowan GJ, Creasey AM, Dhanasarnsombut K, Thomas AW, Remarque EJ, Cavanagh DR (2011). A malaria vaccine based on the polymorphic block 2 region of MSP-1 that elicits a broad serotype-spanning immune response. PLoS ONE.

[26] Galamo CD, Jafarshad A, Blanc C, Druilhe P (2009). Anti-MSP1 block 2 antibodies are effective at parasite killing in an allele-specific manner by monocyte-mediated antibody-dependent cellular inhibition. J Infect Dis.

[27] Ferreira MU, Hartl DL (2007). *Plasmodium falciparum*: worldwide sequence diversity and evolution of the malaria vaccine candidate merozoite surface protein-2 (MSP-2). Exp Parasitol.

[28] Mohammed H, Kassa M, Mekete K, Assefa A, Taye G, Commons RJ (2018). Genetic diversity of the *msp-1*, *msp-2*, and *glurp* genes of *Plasmodium falciparum* isolates in Northwest Ethiopia. Malar J.

[29] Eacret JS, Gonzales DM, Franks RG, Burns JM (2019). Immunization with merozoite surface protein 2 fused to a *Plasmodium*-specific carrier protein elicits strain-specific and strain-transcending, opsonizing antibody. Sci Rep.

[30] Tanabe K, Sakihama N, Walliker D, Babiker H, Abdel-Muhsin AM (2007). Allelic dimorphism-associated restriction of recombination in *Plasmodium falciparum msp1*. Gene.

[31] Rono J, Osier FH, Olsson D, Montgomery S, Mhoja L, Rooth I (2013). Breadth of anti-merozoite antibody responses is associated with the genetic diversity of asymptomatic *Plasmodium falciparum* infections and protection against clinical malaria. Clin Infect Dis.

[32] Muhindo Mavoko H, Kalabuanga M, Delgado-Ratto C, Maketa V, Mukele R, Fungula B (2016). uncomplicated clinical malaria features, the efficacy of artesunate-amodiaquine and their relation with multiplicity of infection in the Democratic Republic of Congo. PLoS ONE.

[33] Mayengue PI, Ndounga M, Malonga FV, Bitemo M, Ntoumi F (2011). Genetic polymorphism of merozoite surface protein-1 and merozoite surface protein-2 in *Plasmodium falciparum* isolates from Brazzaville Republic of Congo. Malar J.

[34] Metoh TN, Chen JH, Fon-Gah P, Zhou X, Moyou-Somo R, Zhou XN (2020). Genetic diversity of *Plasmodium falciparum* and genetic profile in children affected by uncomplicated malaria in Cameroon. Malar J.

[35] Singana BP, Mayengue PI, Niama RF, Ndounga M (2019). Genetic diversity of *Plasmodium falciparum* infection among children with uncomplicated malaria living in Pointe-Noire, Republic of Congo. Pan Afr Med J.

[36] Atroosh WM, Al-Mekhlafi HM, Mahdy MA, Saif-Ali R, Al-Mekhlafi AM, Surin J (2011). Genetic diversity of *Plasmodium falciparum* isolates from Pahang, Malaysia based on MSP-1 and MSP-2 genes. Parasit Vectors.

[37] Mwingira F, Nkwengulila G, Schoepflin S, Sumari D, Beck HP, Snounou G (2011). *Plasmodium falciparum msp1, msp2* and *glurp* allele frequency and diversity in sub-Saharan Africa. Malar J.

[38] Soulama I, Nebie I, Ouedraogo A, Gansane A, Diarra A, Tiono AB (2009). *Plasmodium falciparum* genotypes diversity in symptomatic malaria of children living in an urban and a rural setting in Burkina Faso. Malar J.

[39] Ahmedou Salem MS, Ndiaye M, OuldAbdallahi M, Lekweiry KM, Bogreau H, Konate L (2014). Polymorphism of the merozoite surface protein-1 block 2 region in *Plasmodium falciparum* isolates from Mauritania. Malar J.

[40] Usman-Yamman H, Omalu CJI, Abubakar A, Abolarinwa SO, Eke SS, Otuu CA (2021). Genetic diversity of *Plasmodium falciparum* isolates in Minna, north central nigeria inferred by PCR genotyping of merozoite surface protein 1 and 2. Infect Genet Evol.

[41] Nabet C, Doumbo S, Jeddi F, Konaté S, Manciulli T, Fofana B (2016). Genetic diversity of *Plasmodium falciparum* in human malaria cases in Mali. Malar J.

[42] WHO (2021). Malaria rapid diagnostic test performance: results of WHO product testing of malaria RDTs.

[43] Nankabirwa J, Brooker SJ, Clarke SE, Fernando D, Gitonga CW, Schellenberg D (2014). Malaria in school-age children in Africa: an increasingly important challenge. Trop Med Int Health.

[44] Walldorf JA, Cohee LM, Coalson JE, Bauleni A, Nkanaunena K, Kapito-Tembo A (2015). School-age children are a reservoir of malaria infection in Malawi. PLoS ONE.

[45] Makenga G, Menon S, Baraka V, Minja DTR, Nakato S, Delgado-Ratto C (2020). Prevalence of malaria parasitaemia in school-aged children and pregnant women in endemic settings of sub-Saharan Africa: a systematic review and meta-analysis. Parasite Epidemiol Control.

[46] Coalson JE, Cohee LM, Buchwald AG, Nyambalo A, Kubale J, Seydel KB (2018). Simulation models predict that school-age children are responsible for most human-to-mosquito *Plasmodium falciparum* transmission in southern Malawi. Malar J.

[47] Cohee LM, Opondo C, Clarke SE, Halliday KE, Cano J, Shipper AG (2020). Preventive malaria treatment among school-aged children in sub-Saharan Africa: a systematic review and meta-analyses. Lancet Glob Health.

[48] Nundu SS, Culleton R, Simpson SV, Arima H, Muyembe JJ, Mita T (2021). Malaria parasite species composition of *Plasmodium* infections among asymptomatic and symptomatic school-age children in rural and urban areas of Kinshasa Democratic Republic of Congo. Malar J.

[49] Grobusch MP, Kremsner PG (2005). Uncomplicated malaria. Curr Top Microbiol Immunol.

[50] Day KP, Marsh K (1991). Naturally acquired immunity to *Plasmodium falciparum*. Immunol Today.

[51] Snow RW, Omumbo JA, Lowe B, Molyneux CS, Obiero JO, Palmer A (1997). Relation between severe malaria morbidity in children and level of *Plasmodium falciparum* transmission in Africa. Lancet.

[52] Vafa M, Troye-Blomberg M, Anchang J, Garcia A, Migot-Nabias F (2008). Multiplicity of *Plasmodium falciparum* infection in asymptomatic children in senegal: relation to transmission, age and erythrocyte variants. Malar J.

[53] Contamin H, Fandeur T, Rogier C, Bonnefoy S, Konate L, Trape JF (1996). Different genetic characteristics of *Plasmodium falciparum* isolates collected during successive clinical malaria episodes in Senegalese children. Am J Trop Med Hyg.

[54] Ndiaye T, Sy M, Gaye A, Ndiaye D (2019). Genetic polymorphism of Merozoite Surface Protein 1 (msp1) and 2 (msp2) genes and multiplicity of *Plasmodium falciparum* infection across various endemic areas in Senegal. Afr Health Sci.

[55] Ferrari G, Ntuku HM, Schmidlin S, Diboulo E, Tshefu AK, Lengeler C (2016). A malaria risk map of Kinshasa, Democratic Republic of Congo. Malar J.

[56] Kyei-Baafour E, Tornyigah B, Buade B, Bimi L, Oduro AR, Koram KA (2020). Impact of an irrigation dam on the transmission and diversity of *Plasmodium falciparum* in a seasonal malaria transmission area of Northern Ghana. J Trop Med.

[57] U.S. President’s Malaria Initiative Democratic Republic of the Congo Malaria Operational Plan FY 2022. https://d1u4sg1s9ptc4z.cloudfront.net/uploads/2022/01/FY-2022-DR-Congo-MOP.pdf. Accessed 23 Feb 2022.

[58] Sumari D, Mugasa J, Selemani M, Shekalaghe S, Mugittu K, Gwakisa P (2016). Prevalence of submicroscopic *Plasmodium falciparum* infections in asymptomatic children in low transmission settings in Bagamoyo. Tanzania Malariaworld J.

[59] Yavo W, Konaté A, Mawili-Mboumba DP, Kassi FK, Tshibola Mbuyi ML, Angora EK (2016). Genetic polymorphism of msp1 and msp2 in *Plasmodium falciparum* isolates from Côte d’Ivoire versus Gabon. J Parasitol Res.

[60] Gnagne AP, Konate A, Bedia-Tanoh AV, Amiah-Droh M, Menan HIE, N'Guetta AS (2019). Dynamics of *Plasmodium falciparum* genetic diversity among asymptomatic and symptomatic children in three epidemiological areas in Cote d’Ivoire. Pathog Glob Health.

[61] Lagnika HO, Moussiliou A, Agonhossou R, Sovegnon P, Djihinto OY, Medjigbodo AA (2022). *Plasmodium falciparum* msp1 and msp2 genetic diversity in parasites isolated from symptomatic and asymptomatic malaria subjects in the South of Benin. Parasitol Res.

[62] Huang B, Tuo F, Liang Y, Wu W, Wu G, Huang S (2018). Temporal changes in genetic diversity of msp-1, msp-2, and msp-3 in *Plasmodium falciparum* isolates from Grande Comore Island after introduction of ACT. Malar J.

[63] Mawili-Mboumba DP, Mbondoukwe N, Adande E, Bouyou-Akotet MK (2015). Allelic diversity of MSP1 gene in *Plasmodium falciparum* from rural and urban areas of Gabon. Korean J Parasitol.

[64] Mayengue PI, Luty AJ, Rogier C, Baragatti M, Kremsner PG, Ntoumi F (2009). The multiplicity of *Plasmodium falciparum* infections is associated with acquired immunity to asexual blood stage antigens. Microbes Infect.

[65] Ndiaye T, Sy M, Gaye A, Siddle KJ, Park DJ, Bei AK (2020). Molecular epidemiology of *Plasmodium falciparum* by multiplexed amplicon deep sequencing in senegal. Malar J.

[66] Auburn S, Barry AE (2017). Dissecting malaria biology and epidemiology using population genetics and genomics. Int J Parasitol.

[67] Escalante AA, Ferreira MU, Vinetz JM, Volkman SK, Cui L, Gamboa D (2015). Malaria molecular epidemiology: lessons from the international centers of excellence for malaria research network. Am J Trop Med Hyg.

[68] Nkhoma SC, Nair S, Al-Saai S, Ashley E, McGready R, Phyo AP (2013). Population genetic correlates of declining transmission in a human pathogen. Mol Ecol.

[69] Conway DJ (2007). Molecular epidemiology of malaria. Clin Microbiol Rev.

[70] Anderson TJ, Haubold B, Williams JT, Estrada-Franco JG, Richardson L, Mollinedo R (2000). Microsatellite markers reveal a spectrum of population structures in the malaria parasite *Plasmodium falciparum*. Mol Biol Evol.

[71] Mobegi VA, Loua KM, Ahouidi AD, Satoguina J, Nwakanma DC, Amambua-Ngwa A (2012). Population genetic structure of *Plasmodium falciparum* across a region of diverse endemicity in West Africa. Malar J.

[72] Schneider KA, Escalante AA (2014). A likelihood approach to estimate the number of co-infections. PLoS ONE.

[73] Issifou S, Djikou S, Sanni A, Lekoulou F, Ntoumi F (2001). [No influence of season of transmission nor age of patients on the complexity and genetic diversity of *Plasmodium falciparum* infection in Cotonou, Benin (in French). Bull Soc Pathol Exot.

[74] Babiker HA, Lines J, Hill WG, Walliker D (1997). Population structure of *Plasmodium falciparum* in villages with different malaria endemicity in east Africa. Am J Trop Med Hyg.

[75] Babiker HA, Walliker D (1997). Current views on the population structure of *Plasmodium falciparum*: implications for control. Parasitol Today.

[76] Schoepflin S, Valsangiacomo F, Lin E, Kiniboro B, Mueller I, Felger I (2009). Comparison of *Plasmodium falciparum* allelic frequency distribution in different endemic settings by high-resolution genotyping. Malar J.

[77] McColm AA, Dalton L (1983). Heterologous immunity in rodent malaria: comparison of the degree of cross-immunity generated by vaccination with that produced by exposure to live infection. Ann Trop Med Parasitol.

[78] Legorreta-Herrera M, Ventura-Ayala ML, Licona-Chávez RN, Soto-Cruz I, Hernández-Clemente FF (2004). Early treatment during a primary malaria infection modifies the development of cross immunity. Parasite Immunol.

[79] Collins WE, Jeffery GM (1999). A retrospective examination of sporozoite- and trophozoite-induced infections with *Plasmodium falciparum* in patients previously infected with heterologous species of *Plasmodium*: effect on development of parasitologic and clinical immunity. Am J Trop Med Hyg.

[80] Jeffery GM (1966). Epidemiological significance of repeated infections with homologous and heterologous strains and species of *Plasmodium*. Bull World Health Organ.

[81] Tang J, Templeton TJ, Cao J, Culleton R (2019). The consequences of mixed-species malaria parasite co-infections in mice and mosquitoes for disease severity, parasite fitness, and transmission success. Front Immunol.

[82] Agonhossou R, Akoton R, Lagnika H, Djihinto OY, Sovegnon PM, Saizonou HD (2022). *P. falciparum* msp1 and msp2 genetic diversity in *P. falciparum* single and mixed infection with *P. malariae* among the asymptomatic population in Southern Benin. Parasitol Int.

[83] Ndong Ngomo JM, M'Bondoukwe NP, Yavo W, Bongho Mavoungou LC, Bouyou-Akotet MK, Mawili-Mboumba DP (2018). Spatial and temporal distribution of Pfmsp1 and Pfmsp2 alleles and genetic profile change of *Plasmodium falciparum* populations in Gabon. Acta Trop.

[84] Ogouyèmi-Hounto A, Ndam NT, Kinde Gazard D, d'Almeida S, Koussihoude L, Ollo E (2013). Prevalence of the molecular marker of *Plasmodium falciparum* resistance to chloroquine and sulphadoxine/pyrimethamine in benin seven years after the change of malaria treatment policy. Malar J.

