# Supplementary material for: The diversity of Plasmodium falciparum isolates from asymptomatic and symptomatic school-age children in Kinshasa Province, Democratic Republic of Congo
Source: Malar J. 2023 Mar 20;22:102. doi: 10.1186/s12936-023-04528-z (PMC10025789; doi:10.1186/s12936-023-04528-z)
Supplement: Supplementary file 1 — Additional file 1: Figure S1. Allelic frequencies in pfmsp1 block 2 in asymptomatic (a) and symptomatic (b) carriers and in both rural and urban areas. Figure S2. Allelic frequencies in pfmsp1 block 2 in rural (a) and urban (b) areas. Figure S3. Allelic frequencies in pfmsp1 block 2 in asymptomatic and symptomatic infections stratified by rural (a) and urban (b) areas. Figure S4. Allelic frequencies in pfmsp2 in asymptomatic (a) and symptomatic (b) carriers. Figure S5. Allelic frequencies in pfmsp2 in rural (a) and urban (b) areas. Figure S6. Allelic frequencies in pfmsp2 in asymptomatic and symptomatic carriers stratified rural (a) and urban (b) areas [file 12936_2023_4528_MOESM1_ESM.docx]

**Table S1:** Primer sequences, cycling conditions, and annealing temperatures for *pfmsp1* block 2 and *pfmsp2* nested PCR

|  |  |  |  |  |
| --- | --- | --- | --- | --- |
| **Gene** | **Primer** | **Sequence (5’-3’)** | **Cycling conditions for *msp1* and *msp2*** | **Positive Control** |
|  |  |  |  |  |
| ***msp1*** | M1-OF | CTAGAAGCTTTAGAAGATGCAGTATTG | **Primary PCR:** | 3D7 |
|  | M1-OR | CTTAAATAGTATTCTAATTCAAGTGGATCA | Initial denaturation at 95 °C for 5 min |  |
|  |  |  | followed by 25 cycles of a second |  |
| ***msp2*** | M2-OF | ATGAAGGTAATTAAAACATTGTCTATTATA | denaturation at 94 °C for 1 min, | 3D7 |
|  | M2-OR | CTTTGTTACCATCGGTACATTCTT | annealing at 58 °C for 2 min and |  |
|  |  |  | extension at 72 °C for 2 min, with a |  |
|  |  |  | final extension at 72 °C for 5 min |  |
| ***msp1*** | M1-KF | AAATGAAGAAGAAATTACTACAAAAGGTGC |  | 3D7 |
|  | M1-KR | GCTTGCATCAGCTGGAGGGCTTGCACCAGA |  |  |
|  | M1-MF | AAATGAAGGAACAAGTGGAACAGCTGTTAC | **Nested PCR:** | HB3 |
|  | M1-MR | ATCTGAAGGATTTGTACGTCTTGAATTACC | Initial denaturation at 95 °C for 5 min |  |
|  | M1-RF | TAAAGGATGGAGCAAATACTCAAGTTGTTG | followed by 30 cycles of a second | 7G8 |
|  | M1-RR | CATCTGAAGGATTTGCAGCACCTGGAGATC | denaturation at 94 °C for 1 min, |  |
|  |  |  | annealing at 61 °C for 2 min, and |  |
| ***msp2*** | M2-FCF | AATACTAAGAGTGTAGGTGCARATGCTCCA | extension at 72 °C for 2 min, with a | HB3 |
|  | M2-FCR | TTTTATTTGGTGCATTGCCAGAACTTGAAC | final extension at 72 °C for 5 min |  |
|  | M2-ICF | AGAAGTATGGCAGAAAGTAAKCCTYCTACT |  | 3D7 |
|  | M2-ICR | GATTGTAATTCGGGGGATTCAGTTTGTTCG |  |  |


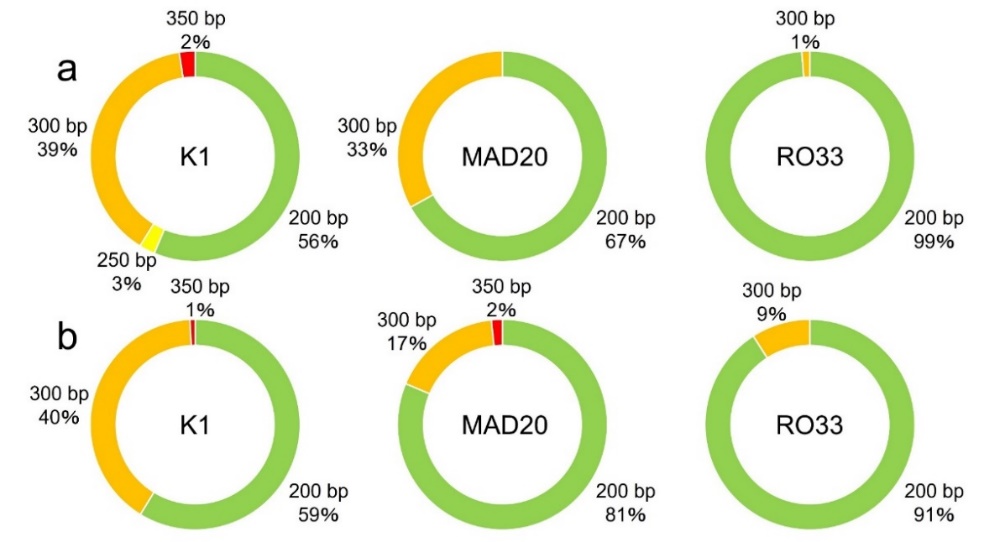


**Fig. S1** Allelic frequencies in *pfmsp1* block 2 in asymptomatic (a) and symptomatic (b) carriers and in both rural and urban areas


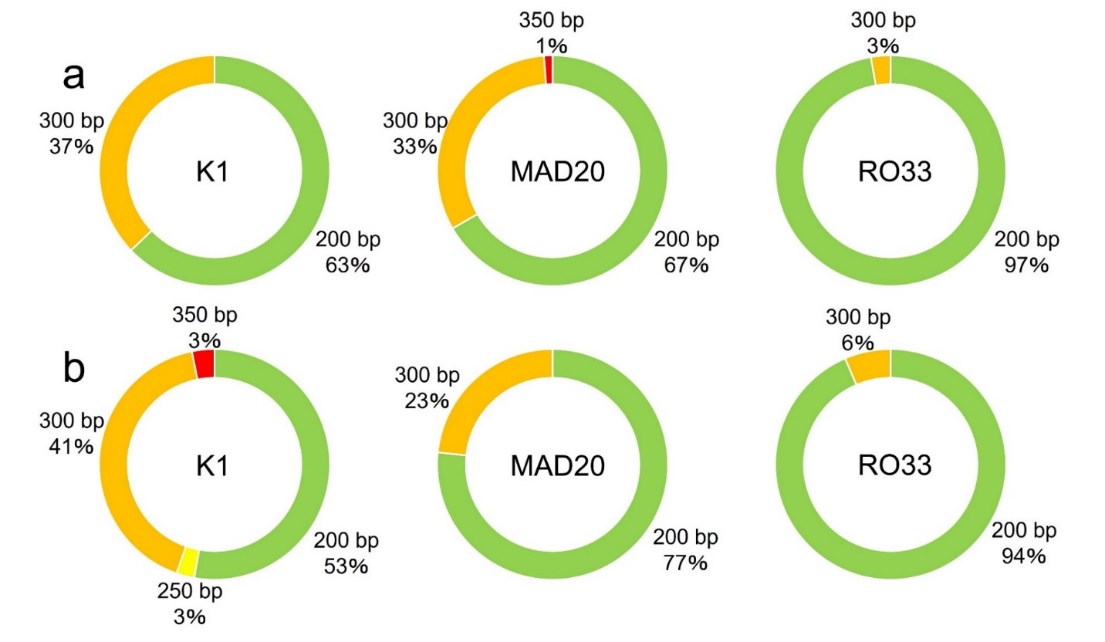


**Fig. S2:** Allelic frequencies in *pfmsp1* block 2 in rural (a) and urban (b) areas


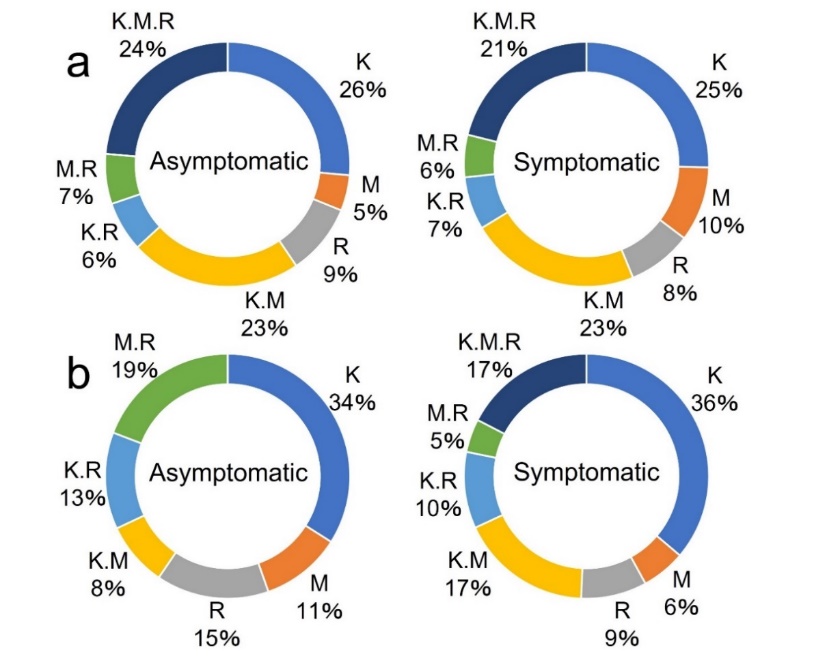


**Fig. S3**: Allelic frequencies in *pfmsp1* block 2 in asymptomatic and symptomatic infections stratified by rural (a) and urban (b) areas


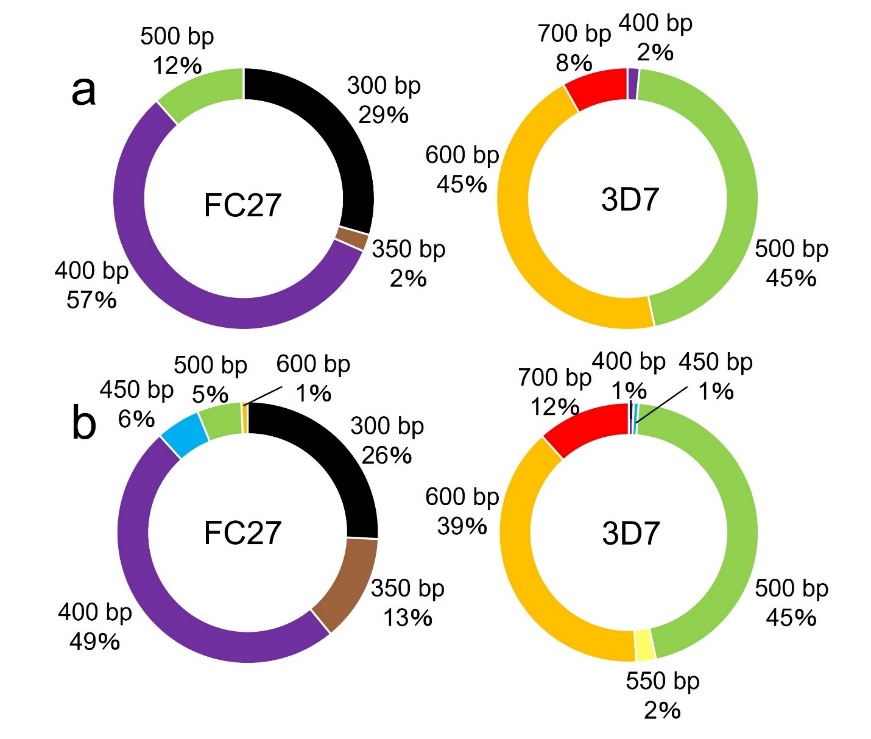


**Fig. S4** Allelic frequencies in *pfmsp2* in asymptomatic (a) and symptomatic (b) carriers


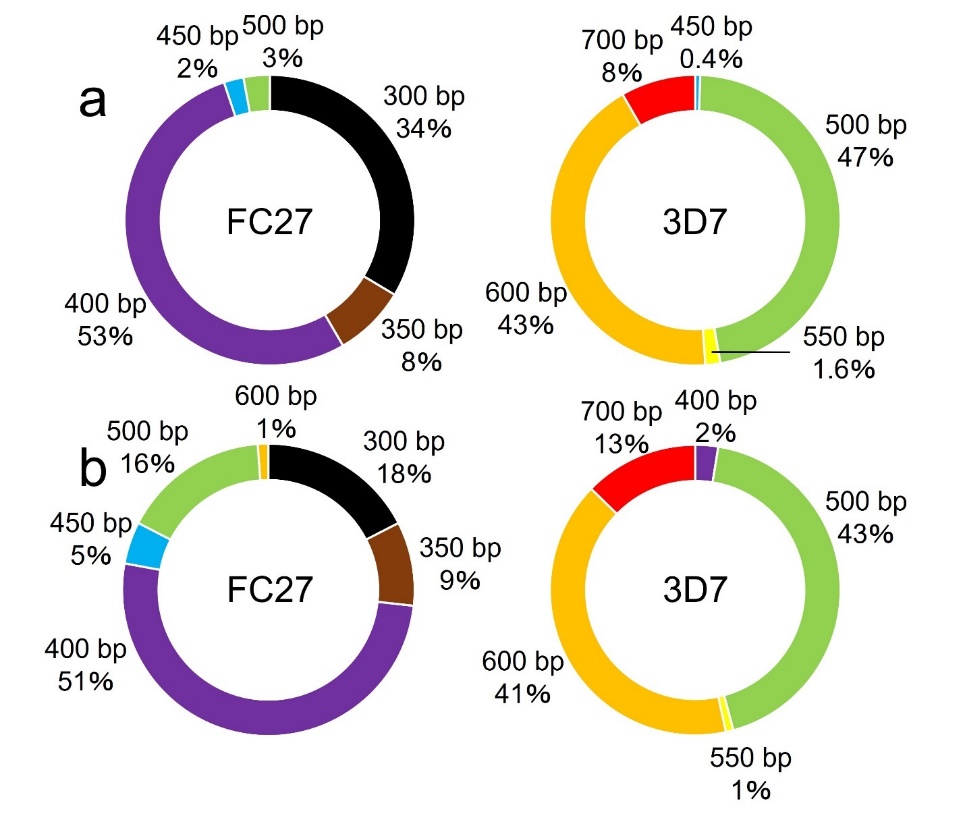


**Fig. S5** Allelic frequencies in *pfmsp2* in rural (a) and urban (b) areas


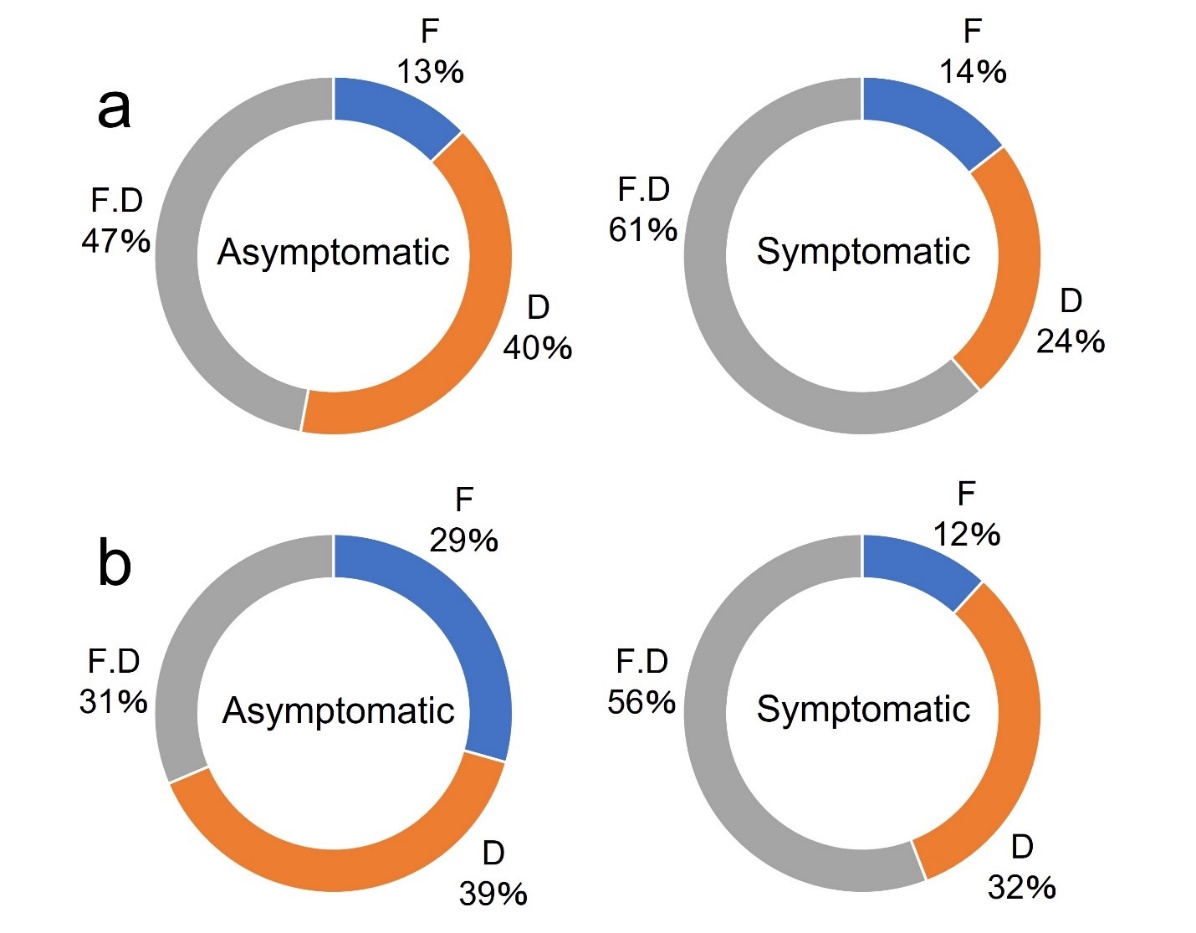


**Fig. S6**: Allelic frequencies in *pfmsp2* in asymptomatic and symptomatic carriers stratified rural (a) and urban (b) areas
